# Ecospirituality and Health: A Systematic Review

**DOI:** 10.1007/s10943-023-01994-2

**Published:** 2024-01-30

**Authors:** Rocío de Diego-Cordero, Alicia Martínez-Herrera, Manuel Coheña-Jiménez, Giancarlo Lucchetti, José Miguel Pérez-Jiménez

**Affiliations:** 1 Research Group PAIDI‑CTS 969 Innovation in HealthCare and Social Determinants of Health, Department of Nursing, Faculty of Nursing, Physiotherapy and Podiatry, University of Seville, Seville, Spain; 2 Department of Nursing, Faculty of Nursing, Physiotherapy and Podiatry, University of Seville, Seville, Spain; 3 Research Group PAIDI-CTS589: Advances in Podiatric Surgery, Department of Podiatry, Faculty of Nursing, Physiotherapy and Podiatry, University of Seville, C/Avenzoar, 6, 41009 Seville, Spain; 4 School of Medicine, Federal University of Juiz de Fora, Juiz de Fora, Brazil; 5 Institute of Biomedicine of Seville (IBiS), Department of Nursing, Faculty of Nursing, Physiotherapy and Podiatry, University of Seville, Seville, Spain; 6 Institute of Biomedicine of Seville (IBiS), Universitary Hospital Virgen Macarena, University of Seville, Seville, Spain

**Keywords:** Ecology, Spiritual, Spirituality, Environment, Health

## Abstract

Environmental changes are affecting human health. A renewal of the way we understand and relate to the planet is needed. Ecospirituality brings together the terms spirituality and environment and is born as a means of solution to this dilemma. This systematic review aimed to find out the influence of ecospirituality on global health. A search of scientific literature was carried out in the main health science databases. A review was conducted to critically evaluate the studies that identified relevant ecospiritual aspects regarding health care for communities. After a systematic search and screening, and following specified methodological criteria, a total of 14 articles were selected in the review. The findings of the review suggest that a new perspective in our worldview such as ecospirituality will provide us with the necessary keys to improve health. To understand ecospirituality, we must keep in mind the indigenous way of life, which is the clear example to follow to achieve environmental health and global health. Ecospirituality leads to a healthier environment, and as this is directly related to health, there is also an improvement in global health.

## Introduction

Negative environmental changes drastically affect society, leading to growing interest in how the environment can affect different individuals and societies around the world. In this context, several areas of interest have focused on human, animal, and plant health and their respective ecosystems. Among these areas, there is growing scientific evidence regarding about the role of spirituality and its connection with the environment in people's physical and mental health (Moreno-Sánchez, 2022).

In modern societies, health professionals must have greater knowledge of the relationship between health and the environment, with the aim of promoting preventive actions. Furthermore, if health professionals increase their knowledge in this area, they can form interdisciplinary teams to discuss environmental policies and collaborate with decision-making processes. Indeed, the health sector needs to be recognized as one of the main pillars of environmental policy, which is why it needs space and skills to work with environmental work sectors, thus being able to know their restrictions and opportunities. In this way, appropriate health promotion, environmental and health services, and workplaces can be guaranteed, making the health sector sustainable from an environmental perspective (Moreno-Sánchez, 2022).

In this sense, renewal of the way we understand and relate to the planet is necessary. In recent decades, we have witnessed a significant environmental crisis, as highlighted by Pope Francis in the second encyclical “Laudato Si” (Bolaños-Sánchez et al., 2015). It is a call for attention to all humanity without making distinctions of religion or race, with the purpose of remembering that we only have one planet on which we can live; and that our permanence on it depends on the respect and care we give it (Villalobos, 2015).

This connection between spirituality and the environment is called “Ecospirituality,” and it is a term used by those facing the current environmental crisis (Lincoln, 2000). It is important to highlight that, although this concept is new, indigenous people have already connected these dimensions for centuries, since their decolonial movement promotes socio-environmental justice, where through their knowledge, beliefs, and styles of inhabiting and caring for the land, approach the term ecospirituality.

**Fig. 1:**
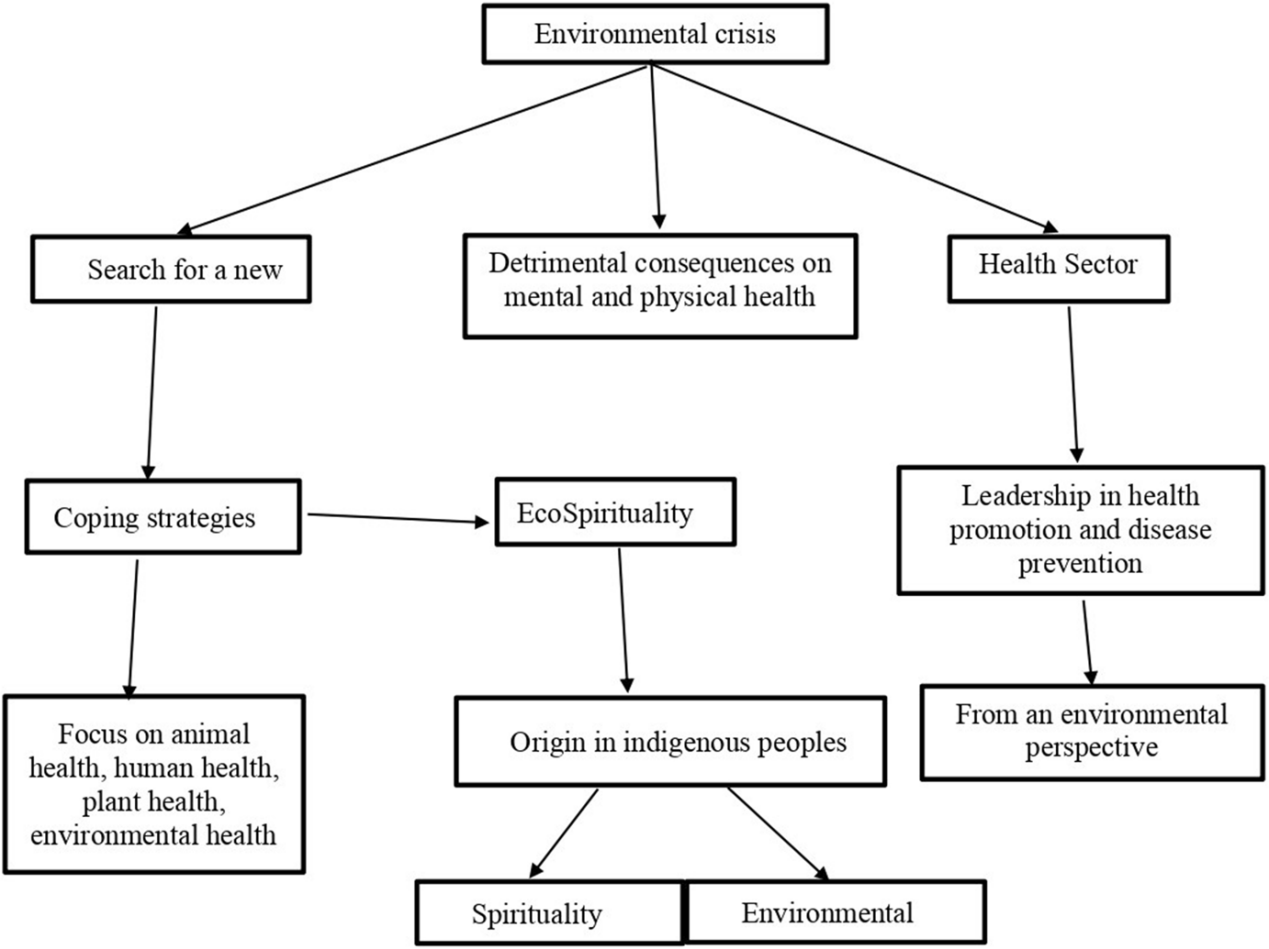
Conceptual framework

The culture prominent in the Western world does not contemplate that human life and non-human life cannot be sustained without each other. It generally supports a materialistic world in which only the accumulation of profits is considered, without considering the reproduction of life. Therefore, we face an environmental crisis that could have a significant influence on health and lifestyle (Silva-de-la-Rosa, 2022). Figure 1 summarizes the conceptual framework used in this review.

This framework, a more holistic view of the environment is offered, covering different aspects for the environment, which includes spirituality. In fact, several researchers have agreed that nature is one facet of spirituality, as seen in the definition of spirituality provided by Puchalski et al. (2009), who consider spirituality as “an essential element of humanity. It encompasses individuals’ search for meaning and purpose; it includes connection with others, with oneself, with nature, and with what is significant or sacred; and encompasses secular and philosophical, as well as religious and cultural, beliefs and practices.” Therefore, it is necessary to put into practice a spirituality that takes nature into account to seek the strategies and skills necessary to take care of each other and survive in the best possible conditions (Silva-de-la-Rosa, 2022).

This review aimed to provide a broader view of the influence of ecospirituality on global health. To this objective, the authors will investigate the relationship of this term within the indigenous population, the repercussions of ecospirituality on the mental and physical health of individuals, the influence of spiritual beliefs in the transitions toward environmental sustainability, and the ecospiritual initiatives designed for health promotion.

## Methods

### Study Selection and Eligibility Criteria

Studies with qualitative/quantitative/mixed experimental designs that considered ecospirituality and health were included. The eligibility criteria for this review were as follows: The inclusion criteria were articles published in the past 5 years (between 2018 and 2023) and available in English and Spanish. Articles published in other languages, not subjected to peer review, were only available in Abstracts and not assessing the objectives of this review were excluded.

### Quality Appraisal

The quality of the studies was appraised using the Mixed Method Appraisal Tool (MMAT) (Hong et al., 2018). Developed to the assess the methodological quality of empirical studies, the latest version of MMAT includes two screening questions and five criteria for each of the following five study designs (Hong et al., 2018). The study designs were as follows: (a) qualitative; (b) randomized controlled trial; (c) nonrandomized; (d) quantitative descriptive; and (e) mixed methods studies. An overall score of the quality appraisal of each study and a detailed appraisal are presented.

### Search Strategy

A literature review was conducted between February and May 2023. The PubMed, Scopus, and Web of Science databases were used for this review. The search strategy was designed based on the DeCS/MeSH descriptors, and terms were combined using the Boolean operators "AND" and "OR" as follows in Table 1.

**Table 1:** Search strategy

Database strategy	CINAHL (from 2018 to 2023) #1 AND #2
1	(spiritual*[All Fields] OR ecospirituality [All Fields] OR "spiritualism"[MeSH Terms] OR "spiritualism"[All Fields] OR "spirituality"[MeSH Terms] OR "spirituality"[All Fields] OR "spiritualities"[All Fields] OR "spirituality s"[All Fields] OR "spiritually"[All Fields] OR "spirituals"[All Fields])
2	("ecolog*"[All Fields]) (environ*[All Fields] OR "environment"[MeSH Terms] OR "environmental"[All Fields] OR "environmentalist"[MeSH Terms] AND ("spiritual*"[All Fields])
Limiters applied	English language, peer-reviewed
Search results on 30/05/2023	

### Study Selection and Data Extraction

After the search was performed searching by two independent researchers, all references were imported using the Mendeley reference manager version 1.19.8. Any disagreements regarding inclusion were resolved by a third researcher. In this review, the sources of information are classified into primary sources (close to the topic investigated, that is, the most direct information without the existence of intermediaries, such as surveys or interviews), secondary sources (based on the primary sources, there are intermediaries), synthetic, analytical, or reorganizing sources (e.g., reviews or biographies), and tertiary sources (compilation of primary and/or secondary sources and their comments on the subject, e.g., guides or indexes) (Cruz-García, 2019). Then, a thematic categorization was carried out with the main findings identified during the analysis, which was carried out through discussions by the researchers, who examined themes or patterns of meaning within the data and grouped the studies into themes. The obtained themes are described in the next section of the manuscript.

## Results

**Fig. 2:**
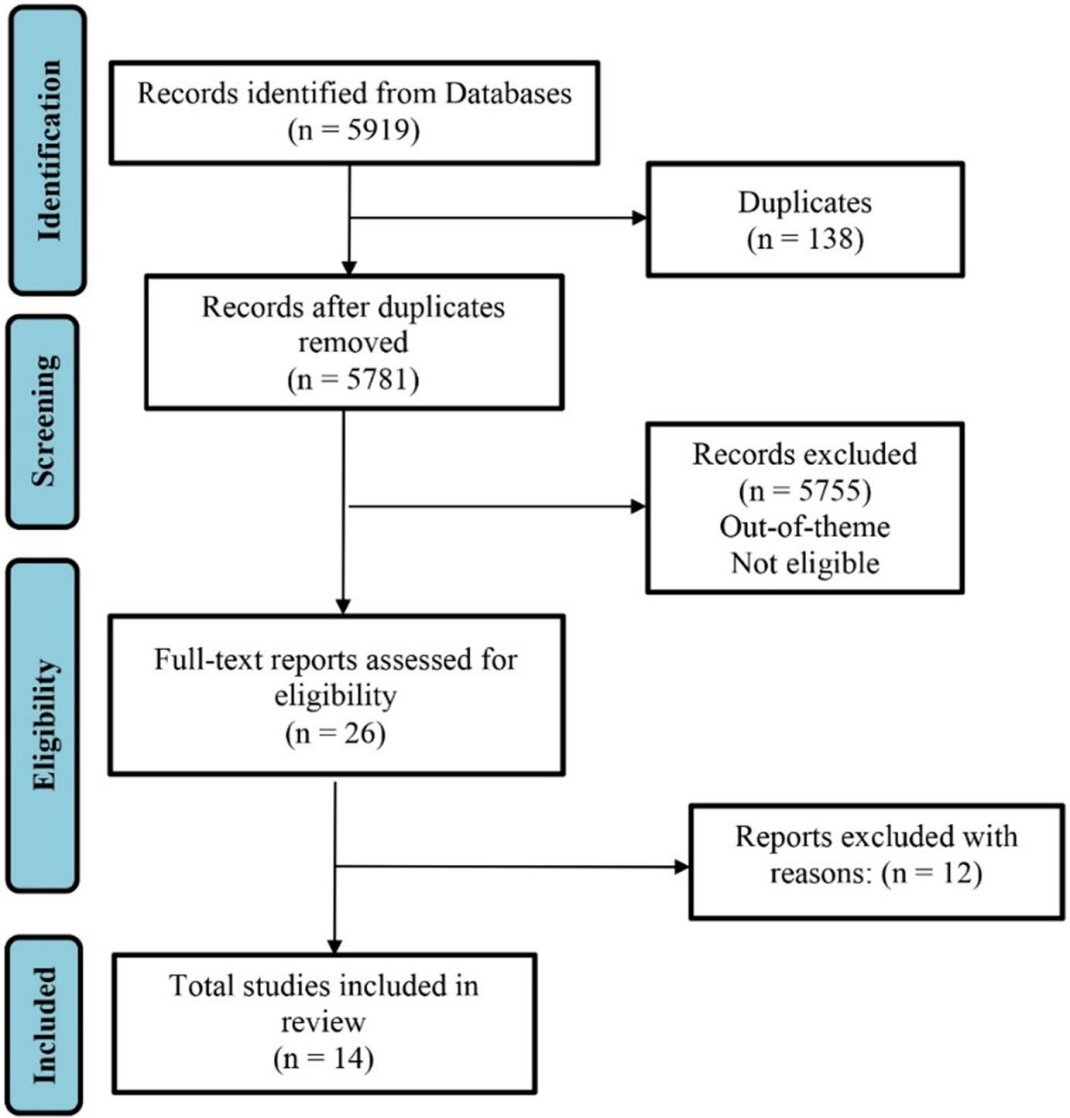
Flow diagram of screening

A flowchart was developed according to the PRISMA Statement (Page et al., 2021). First, 5919 articles were retrieved from the databases using Boolean expression. After eliminating duplicate records (*n* = 138), it remained 5781 references, which were assessed through the title and abstract. Among these references, 26 were eligible for full-text reading, while the others were out-of-theme or not adherent to the inclusion/exclusion criteria. No results were obtained from the gray literature search. Full-text reading of the 26 references resulted in the inclusion of 14 studies, of which: Four were case studies, seven were literature reviews, one was a cross-sectional study, one was a longitudinal study, and one was a mixed method study (Fig. 2).

### Result of Quality Appraisal

Appendix 1 outlines the results of the MMAT quality appraisal of the individual studies. One study was identified as a mixed method (Nawrath et al., 2022), nine as qualitative studies (Bellehumeur et al., 2022; Lestar & Böhm, 2020; Cloud & Redvers, 2023; Denis et al., 2022; Hategan, 2021; Keaulana et al., 2021; Skoko et al., 2021; Nalau et al., 2018; Oberholzer-Dent et al., 2023), and four as quantitative studies (Kras & Keenan, 2020; Oliveira et al., 2022; Vitorino et al., 2018; Walls et al., 2022). All included studies addressed both screening questions, stated the research aims, and justified the findings to answer the research questions. Overall, all studies met three or more of the five quality appraisal criteria of the MMAT.

**Fig. 3:**
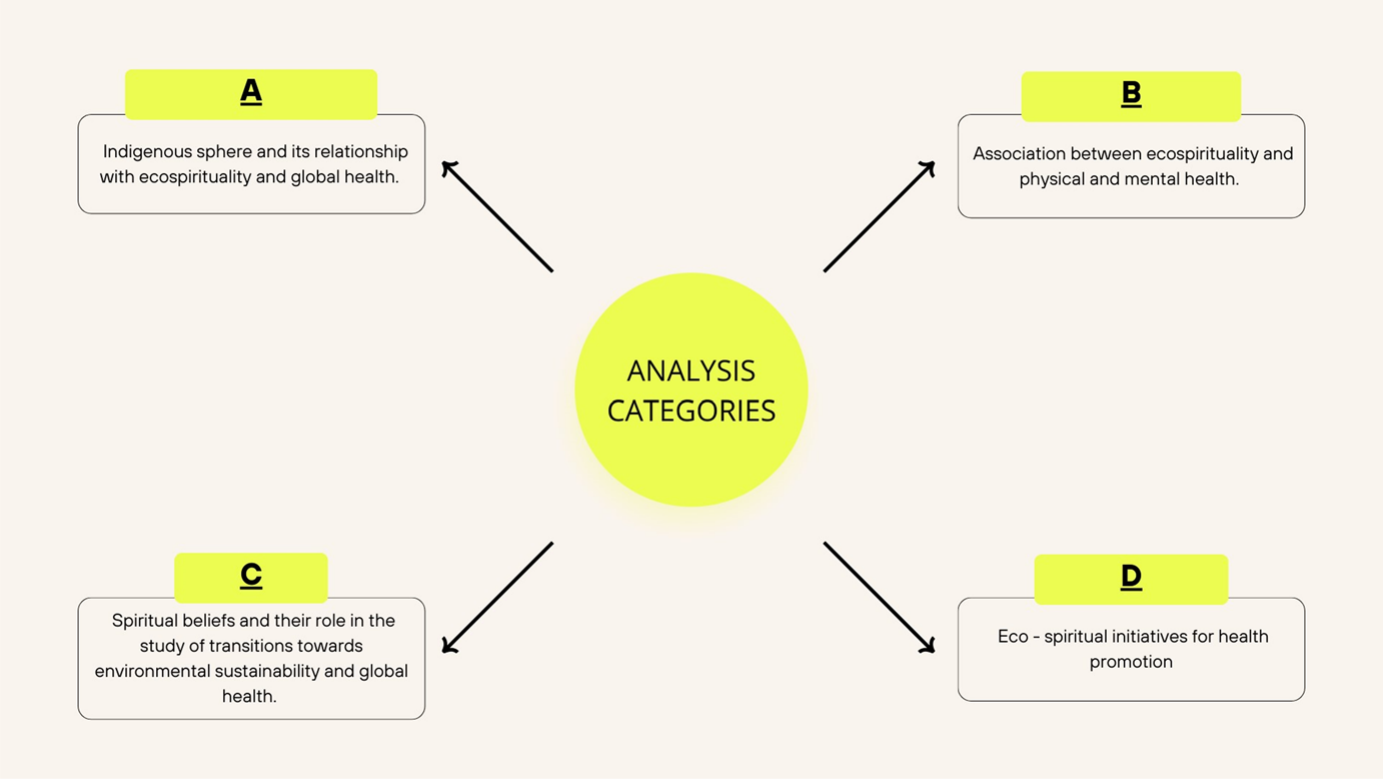
Coding tree with the main categories

The thematic categorization resulted in the following categories shown in Fig. 3: Category A: Indigenous sphere and its relationship with ecospirituality and global health. Category B: Association between ecospirituality and physical and mental health. Category C: Spiritual beliefs and their role in the study of transitions toward environmental sustainability and global health. Category D: Ecospiritual initiatives for health promotion. An analysis and synthesis table was also prepared with the essential information for each result included (Table 2).

**Table 2:** Results

Author year/country	Design and sample	Aims	Main results/categorization of results
Oberholzer-Dent et al. (2023)/EEUU	Study of cases	Research the contemporary work of the California Indian Basket Weavers Association and demonstrate how indigenous environmental justice is put into practice	Addressing environmental management from an indigenous perspective obtains multiple environmental benefits and, in turn, improves the health of individuals./A
Kras and Keenan (2020)/EEUU	Study of cases	Understand how the natural environment influences the lives of adult residents living on these entire New England islands and guide future research	The natural environment plays a vital role in the lives of the inhabitants of the New England Islands, causing an improvement in their mental health./A, B
Vitorino et al. (2018)/EEUU	Transversal study	Investigate how different levels of spirituality and religiosity affect quality of life and mental health	Having high levels of both spirituality and religiosity is associated with a better quality of life (psychological, social, and environmental)./B
Nalau et al. (2018)/Nueva Zelanda	Study of cases	Determine how indigenous and traditional knowledge relates to climate change adaptation and ecosystem-based adaptation	Recognizing indigenous governance systems and indigenous and traditional knowledge will give us a different vision in our connection with nature, thus improving health./A
Lestar and Böhm (2020)/Inglaterra	Literature review	Defend the inclusion of spirituality, as a worldview, in the study of transitions toward sustainability	Ecospiritual communities are a mechanism to address health problems and reduce negative consequences of the social determinants of health due to better treatment with the natural environment./C
Bellehumeur et al. (2022)/Canadá	Literature review	Explore the relationship between humans and nature through positive psychology	A new "consciousness" is emerging in which people define their quality of life in spiritual and relational rather than materialistic terms./B, C
Skoko et al. (2021)/Croacia	Literature review	Demonstrate that religious people cope better and more easily with the dangers that can damage mental health	Religiosity appears to be a crucial factor in prolonging human life and greater resistance to some diseases./B
Denis et al. (2022)/Francia	Literature review	Investigate a systemic approach that integrates the biological, psychological, and social systems that can influence health or disease	The biopsychosocial model is the most precise theoretical and clinical model of health and illness that we currently have./D
Walls et al. (2022)/EEUU & Canadá	Longitudinal study	Examine seven different indicators of sociocultural integration and identify patterns of responses across these indicators and determine their relevance to well-being	The connection between humans and the sense of belonging to a group and to nature influences better physical and mental health./C, D
Keaulana et al. (2021)/EEUU	Literature review	Identify and summarize existing measures of connectivity, relatedness, and attitudes toward land, nature, and/or the environment and evaluate the psychometric properties of these scales	Increasing connections with nature, the land, and the broader environment can improve the overall meaning of life and human well-being./A, C
Hategan (2021)/Rusia	Literature review	Investigate trends: ecophilosophy, ecotheology, and counseling to apply them in the community	The use of ecological advice in practice allows people to generate new strategies to face the environmental crisis and thus motivate them to change their lifestyle for a healthier one./D
K	Literature review	Expand understanding and connectivity between indigenous traditional knowledge, sacred places, spirit, and environmental health	The connection with nature and the environment improves health in all its aspects, in addition to attitudes and behaviors./A
Oliveira et al. (2022)/Brasil	Study of cases	To investigate the influence of religiosity and spirituality on the adoption of prophylactic behaviors and the perception of risk of vulnerability to the disease	More religious/spiritual people more frequently adopt prophylactic behaviors./C, D
Nawrath et al. (2022)/Reino Unido	Sequential mixed methods study	Explore the perception between mental health and green spaces of a population of low socioeconomic levels	Green spaces can play a fundamental role in increasing mental health in low socioeconomic populations./B

## Discussion

This study aimed to critically review the current evidence regarding the influence of ecospirituality on global health. The findings of the review highlighted the repercussions of ecospirituality for the mental and physical health of individuals and the influence of spiritual beliefs in the transition toward environmental sustainability. The synthesis of key findings from the 14 included studies was summarized in four categories.

### Category A: Indigenous Sphere and Its Relationship with Ecospirituality and Global Health

The results included in this review show how colonialism has had an impact on the current environmental crisis and argue that, in the future, the knowledge of indigenous individuals must be taken into account to establish links with the environment. Indigenous peoples are highly vulnerable to the negative impacts of climate change given their dependence on climate-sensitive resource-based livelihoods. This is aggravated because their financial, technological, and political resources are very limited, being on the margins of the prevailing capitalist society, which prevents them from reacting and making their needs known to rulers. Despite this, they have historically demonstrated their ability to adapt to climate and social, cultural, political, and economic changes. This resilience can be used as a powerful tool to assist in the implementation of future environmental projects for this population (Nalau et al., 2018). It is very important to give indigenous populations the value they deserve and to offer proposals for the protection of life and the great biodiversity they have managed to preserve, despite the complex scenario of climate change.

Because of colonization and uncontrolled environmental management, indigenous people today face many obstacles. Ecosystem degradation, settler management of indigenous lands, herbicides and pesticides, competition with commercial collectors, and inadequate waste disposal have destroyed the ecosystems in which these communities operate. Indigenous environmental justice (IAJ) understands environmental harm and proposes relationships with the environment through indigenous vision. Considering that the colonization process and the environmental crisis are directly related, the authors want to achieve the authority of indigenous peoples and the survival of this environmental catastrophe through the vision of justice in indigenous terms. The current environmental crisis recognizes the importance of indigenous communities and their relationship with the land to address this critical aspect, thus improving health. To understand the equitable and accurate use of natural resources, one must first understand the cultures that have developed based on this idea (Oberholzer-Dent et al., 2023). A paradigm shift focusing on rights and equality is needed, with inclusion being the fundamental principle for closing structural inequality gaps. In this sense, ecospirituality is a tool that combines ecospirituality.

Knowledge of indigenous peoples establishes an important conception of the vision of the world and environment. Each of the existing indigenous peoples has a specific and distinct norm rooted in nature. The knowledge of indigenous peoples and their spiritual practices is related to ancestral and traditional territories represented by sacred places. Indigenous communities have the exclusive ability to adapt and to connect with their natural environment. In sacred places, indigenous peoples connect with their ancestors, nature, and even the universe, causing a commitment of these peoples to environmental health. However, these sacred places are threatened by colonization and environmental crises. These spaces carry thousands of years of traditional ecological and spiritual knowledge, which can disappear. In the majority of indigenous communities, nature has a spirit; therefore, spiritual knowledge is equivalent to the knowledge of the natural environment. Therefore, to achieve environmental health, it is necessary to understand and value the spirit present in all the elements of the environment. In this way, understanding the indigenous spiritual realm with the environment will make humanity appreciate nature and connect it with it. Indigenous people express their knowledge through how they perceive and relate to the world. The relationship with nature must include an exchange in which every time the environment gives us something, we give it back in another way, thus establishing a spiritual connection. The belief of many indigenous people is that everything in the universe is related and that is why all “energy” comes and goes forming a circle (Cloud & Redvers, 2023).

The previous studies on climate adaptation based on indigenous traditional knowledge (ITK) used but did not integrate the indigenous population in their research. That is, they simply extracted information for their research projects, and the indigenous communities did not receive follow-up on the findings obtained, climate adaptation projects, or funding to facilitate climate adaptation, thus generating discontent among these individuals, who were reluctant to share certain information in other studies. In research on the indigenous populations, it must be considered that the tendencies of men and women can differ greatly due to the main subsistence strategies; hence, it is important to consider gender differences. Men and women have different types of ITK, which, in turn, impact the types of livelihoods they practice and the knowledge they use in those activities. Therefore, when investigating ITKs, the entire community, as well as men and older people, must be considered (Nalau et al., 2018).

In this sense, research, such as that of the inhabitants of the New England Islands, has contemplated what happens when a population lives isolated from the people of the west, presenting a lifestyle that is more similar to that of indigenous communities. The islanders lived in natural places where they depended on nature for fundamental life activities. This community stated that there is a feeling of being separated from the rest of the world and that is has positive and negative sides. The positive aspect is that they depend on themselves and reinforce the unity of the community despite the challenges of climate, transportation, and limited commercialization. Furthermore, the natural spaces were preserved because of this insolation. The negative aspect is the concern about feeling totally at the mercy of Mother Nature. The permanent inhabitants of the New England Islands enjoyed the isolation they came with living there. They even expressed great gratitude and adoration for the island's natural ecosystems, in addition to expressing concern for the environment, due to tourism during the summer months. Furthermore, the natural environment has been shown to have a positive impact on many aspects of life, such as physical exercise, mental health, and spiritual interconnection with nature (Kras & Keenan, 2020).

Incorporating indigenous worldviews about nature helps humanity to improve our understanding of ecospirituality. The connection between nature and the environment improves health in all aspects, in addition to attitudes and behaviors. Once again, the indigenous community stands out as a representative of the ecospiritual movement for its possession of knowledge and values that are based on a close bond with nature. They demonstrate the link between nature and improved health at all levels. With ecospirituality understood from the indigenous sphere, environmental health can be achieved, thereby achieving global health in populations as a direct consequence (Cloud & Redvers, 2023; Keaulana et al., 2021). The cosmovision of indigenous peoples can illuminate our ecospirituality, something that the capitalist and extractivist systems will never understand.

In this regard, the previous studies have indicated that the defensive nature of colonialism has influenced the desolation of nature and, with it, the ruin of the most unprotected in society. It is proposed to investigate the models of colonial socio-environmental domination in which capitalist spirituality is obligatorily established with the purpose of ending this thinking and assimilating the importance of decolonial resistance to obtain socio-environmental justice. For this reason, they position themselves in favor of a spirituality that strengthens the bond between humans and nature: ecospirituality (Silva-de-la-Rosa, 2022). The self-demarcation of the territory of indigenous populations is spiritual, it is what their ancestors have left them. Despite this lack of ownership, indigenous territories are considered the best preserved on the planet.

Other studies have reported the need for a different formulation of the JIA to address the ecological crisis, considering indigenous conceptions of what justice includes, both in the theoretical framework and when taking real actions. Indigenous nations have recognized the main problems of the ecological crisis and have proposed their own solutions: The environmental crisis must be considered an intensification of colonialism; therefore, a decolonization process is essential to solve the problem (McGregor et al., 2020).

Recently in 2020, another study clarified that indigenous populations do not include the concept of ecospirituality in their vocabulary because there are certain current words that are not part of their language. However, their way of life, and their values and principles indicated that they put this concept into practice, which is how indigenous peoples were considered leaders in this concept, and without them having named the word (Ecospirituality churches workgroup and Mining Network, 2020).

### Category B: Association Between Ecospirituality and Physical and Mental Health

The results of the studies included in this review indicate that ecospirituality promotes behaviors in favor of the environment and mental health. To establish an association between ecospirituality and mental health, firstly, the authors analyze how nature and spirituality independently influence mental health. Mental disorders are becoming a global problem and are increasing dayly. In low- and middle-income countries that have undergone a rapid urbanization process, these types of cases are increasing the most. A series of factors promote the development of mental disorders, such as a decrease in physical exercise and an increase in violence, poverty, social isolation, overcrowding, and air and noise pollution.

The previous studies have pointed out the positive relationship between nature and mental health, which creates a problem for the population living in cities who spend less time exposed to the natural environment. Even so, it must be considered that the different historical and cultural contexts of the different populations influence this positive relationship, being greater or lesser depending on the sociocultural characteristics of the community. Even so, it must be considered that the different historical and cultural contexts of different populations influence this positive relationship, being greater or lesser depending on the sociocultural characteristics of the community. Green spaces encourage physical activity and social cohesion, which reduces mental health problems (Nawrath et al., 2022). Environmental crises are known to have consequences on physical and health, social well-being, and mental health for both the short and long term.

The relationship with nature can be experienced from two perspectives, considering it as a medium that evokes intense positive feelings and connection, or considering it as a threat when perceiving the diminution of the Self within the immensity of the Universe. Most studies have demonstrated the physical, mental, and behavioral benefits of being in contact with the natural environment. However, studies indicate that exposure to natural disasters or knowledge about them generates significant stress or anxiety in some individuals. This situation can generate frustration and helplessness owing to the inevitable consequences of the environmental crisis, both in the short and long term (Bellehumeur et al., 2022). Similarly, other studies have explained how the natural environment prevents or helps against certain mental disorders such as depression, anxiety, and substance abuse. This demonstrates a close link between mental health and environment (Kras & Keenan, 2020).

On the other hand, as other studies have pointed out, some German philosophers studied the relationship between psychosomatic illnesses and theological anthropology. The authors concluding that these illnesses cannot be genuinely treated without also attending to the spiritual dimension. They stated that the body is an expression of the soul, and that illness appears in the mental area when the individual is unable to comply with him or her conscience demands, and cannot establish a deep bond with himself. They stated that most somatic illnesses were due to problems at the spiritual level. This means that the areas of theology and psychology are interconnected, and the interconnection between them is spirituality that establishes harmony between them. Trying to solve a mental problem without a therapy that focuses on spirituality will not provide a permanent solution and will make the patient always dependent on their therapist. According to Skoko et al. (2021), spirituality has become a decisive factor in achieving good mental health, improving adaptation to illness, and prolonging life. It must be taken into account that spirituality does not protect people from illness, but it does have a positive influence on a psychological and physical level, thus becoming a strategy to face and adapt to an illness (Skoko et al., 2021). These approaches coincide with the fact that high levels of spirituality provide a better quality of life at the mental, social, and environmental levels (Vitorino et al., 2018).

Furthermore, spending time in contact with nature, seen as a spiritual practice, provides humanity with a connection with the planet, evokes healthy behaviors for the environment, and therefore for people's mental health. By integrating spirituality within the framework of climate change and nature, the term ecospirituality could begin to be used, since the purpose of what is explained is to exalt the value of everything that makes up nature: human and non-human, and to guide our actions to protect the planet and ourselves (Bellehumeur et al., 2022). Encouraging the encounter between the practices of ecospiritualities could help to bring greater strengthen the struggles for environmental justice in communities.

In this regard, the previous studies corroborate that urbanization causes sedentary lifestyles and unhealthy eating, which favor mental disorders. Therefore, it has been proposed that urban areas maintain a balance with the existence of green spaces. Furthermore, it is once again argued that contact with nature favors the promotion of health and prevention of both physical and mental illnesses, and this connection can become a therapeutic and rehabilitative measure (Castell, 2020; Martínez-Soto et al, 2014). Other studies have highlighted the significant relationship between spirituality and mental health, concluding that the more spirituality is cultivated and encouraged, the greater the perception of psychological well-being and the greater the mental health of the individual (Cano-García & Quintero-Núñez, 2020).

### Category C: Spiritual Beliefs and Their Role in the Study of Transitions Toward Environmental Sustainability and Global Health

Research findings suggest that ecospirituality directs humanity toward a healthier environment, and since the environment is directly related to health, there is also an improvement in overall health. Other studies have supported a relationship between environmental problems and human health. Specifically, at a global level, concerns about the challenges presented by environmental crises are increasing every day (Bellehumeur et al., 2022; Lestar & Böhm, 2020).

In response to this problem, technological solutions such as electric cars, the use of public transport, and biofuels have been proposed and implemented. With these methods, it is assumed that sociotechnical regimes can be maintained using low-carbon technologies, and not only does this serve as a remedy for the environmental crisis. To achieve this, it is also important to change human behaviors and practices by creating values and belief systems aimed at the sustainability of the planet, which implies opposition to capitalist growth. In these reflections made by professionals in the ecological field, consideration of spirituality and religion is usually missing. Consequently, the term ecospirituality is proposed as a way of seeing the world to include it in debates about sustainability. Ecospirituality is a very broad term that includes many ecological theories and practices, emphasizing the successes of the practical field and understanding it as any action in favor of the health of the environment or lifestyles that represent actions in favor of the environment. It could be understood as a “pro-environmental worldview,” where the beliefs and assumptions of this vision lead to attitudes and behaviors that are beneficial to the environment.

Therefore, ecospirituality usually advocates the following lifestyles: vegetarianism, a close connection with nature and all its beings, prudence and simplicity, sharing, creativity, and meaningful manual work. In the concept of ecospirituality, Earth is considered a place whose objective is to bring happiness to all living beings. Gone is the view that humans “dominate” animals and nature, which is why they support or force a change in diet, which is one of the fundamental pillars in the transition toward environmental sustainability (Lestar & Böhm, 2020). Ecospirituality increases ecological and food education and produces creative, versatile, and satisfying work environments, as well as mechanism to address health problems and reduce the negative consequences of the social determinants of health (Keaulana et al., 2021; Lestar & Böhm, 2020).

There are various research findings in parallel with this view, the study of Oliveira et al. (2022) revealed that ecospirituality promotes a series of prophylactic behaviors and a greater perception of vulnerability to diseases. It can be said that ecospirituality can guide humanity toward a healthier life at the environmental and global health levels. The previous studies have suggested that there is a clear link between nature and the environment with improved health at all levels; therefore, the connection between health and well-being with nature and the environment must be considered (Keaulana et al., 2021).

The world is increasingly aware that the current economic system stimulated by technology does not serve to solve the environmental crisis, which is why it is necessary that social factors, including ecospirituality, be included in ecological research and practices (Lestar & Böhm, 2020). In this regard, the previous studies have indicated that the concept of health must address all areas of the human being, including the spiritual, as well as the environment and its link with the beings of the planet. The aim is to leave aside the individual vision of health and illness and the doctor–patient relationship, and broaden the perspective to a social level, since global health implies the action of the entire population in an open and changing space (Hurtado et al., 2021). The lack of respect for ecology is similar to or can be an expression of social unconsciousness, as it affects not only individuals but also social, political, and cultural structures. However, it also concerns all of humanity because in our daily lives, we adopt attitudes that have a positive and negative impact on the environment. Spirituality favors ecological conversion. A change in mentality, the adoption of individual attitudes, and participation in collective and institutional actions will help protect our planet.

Other studies claim that delving into people's spiritual spheres reduces stress and, therefore, related diseases. Furthermore, they consider that the cultivation of spirituality provokes emotional learning, causing the individual to be able to detach from the material and approach what is truly important, finding a positive meaning in the illness or in any unfavorable event in the person's life (Sarrazin, 2021). Without forgetting, Pope Francis' second Encyclical “Laudato Si” reflects on how humanity is awakening its conscience toward a greater bond of respect with the environment and nature. It is not too late to establish a change and rectify the mistakes committed against nature (Villalobos, 2015).

### Category 4: Ecospiritual Initiatives for Health Promotion

The findings obtained from the review indicated the importance of the health sector using a biopsychosocial model for its care, where work is done in the spiritual sphere and through it strategies based on ecospirituality are applied, such as sociocultural integration and ecocounseling. A biopsychosocial approach to the individual allows to study the person as a whole and stop considering only the physiological part. In addition to analyzing the person, we must take into account the great influence of the social and natural environment where they develop, so maintaining or improving health does not depend exclusively on individual movement (Denis et al., 2022).

Therefore, what is needed is an open mind about how an individual's particular symptoms shape their biology. Furthermore, each patient is unique and, therefore, needs to actively participate through the perception of their health status. Without forgetting some basic fundamentals such as that people with lower socioeconomic levels are more likely to get sick and die younger. Differences in religion, gender, race, or age suggest that in today's world, not all people have the same opportunities to live a long and healthy life. There are also elements in the individual's environment that increase the risk of getting sick, such as chemicals, combustion emissions, or radioactivity. Patients living with diseases need health professionals to take “traditional” measures in charge of modifying physiological parameters. In addition, interventions based on psychosocial factors must be included, since people's beliefs and expectations directly influence the result of treatments more than humanity think. A therapeutic relationship is needed that includes interventions and relational skills in a holistic way. Therefore, the biopsychosocial model is the most correct model of health and disease, despite its limitations in relation to the number and variety of health determinants. By intervening with this model, interventions could be carried out in the spiritual field, where the clinicians would include the ecospiritual vision for our patients (Denis et al., 2022). In this sense, the previous studies between chronic diseases and spirituality have suggested that the training and awareness of health professionals in the spiritual field is necessary to be able to treat people with chronic diseases (Skoko et al., 2021).

As a first initiative, sociocultural integration is valued, which consists of an expanded evaluation of the social and cultural aspects of relevance and connection. The connection between humans and the sense of belonging to a group and to nature influences better physical and mental health. This integration is experienced in different ways depending on the cultural community to which one belongs, due to the system of values and beliefs that comprise it. The theories and models of indigenous peoples encourage relationships between individuals, families, communities, nature, spirit, and culture, from which we can better understand sociocultural integration. Within health promotion, space must be left to promote sociocultural integration, through the promotion of cultural identity and the links between people and nature. To achieve this, they must be based on indigenous worldviews, through an ecospiritual perspective, where within their culture emphasis is placed on the importance of interconnections and the creation of links with individuals, families, communities, nature, the spirit, and culture (Walls et al., 2022).

Another initiative would be the use in practice of the concept called eco-counseling, which consists of preventing and addressing life problems and conflicts related to life crises through an approach that integrates the interaction of personal and environmental factors. This focus empowering individuals for self-help and initiative, making them responsible for their actions, since the healthy individual is taken and what is sought is to enhance their capabilities. Ecology gave rise to the concepts of ecophilosophy and ecospirituality. These two concepts can help establish a new vision in modern society. The ecological trend that unites these terms is responsible for resolving the situations corresponding to the environmental crisis. Using these terms with a spiritual approach through eco-counseling in the health sector benefits the community. By addressing it through ecophilosophy and ecospirituality, it seeks to develop new skills and abilities in the community following an ecological basis (Hategan, 2021).

The previous studies indicate that counseling causes greater success at a lower cost for the health professional who implements it, in addition to making the patient show more security and motivation to change their lifestyle to a healthier one. Even health institutions benefit from this technique because it increases the quality of care perceived by patients and improves working relationships between health professionals. The final beneficiary of the practice of counseling turns out to be the society that ends up adopting healthier behaviors (Bimbela, 2009).

On the other hand, as established in the Code of Ethics of the International Council of Nurses (ICN) in the element “Nurses and global health,” nurses examine the importance of the social determinants of health and act on them, coming from health programs. Furthermore, they recognize the impact of the natural environment on human health and, therefore, support any decision that reduces practices harmful to the environment (International Council of Nurses, 2021). This approach suggests the incorporation of ecospirituality in the daily practice of health professionals.

### Recommendations for Clinical Practice

The following recommendations can be drawn from the rapid review. In 2013, Pope Francis issued an encyclical letter called Laudato si, which urges the global community to take care of the environment as a concern shared by all. Pope Francis discussed the various outcomes of scientific progress and acknowledged its contributions to human advancement. Nonetheless, he emphasized the need for humanity to recognize the limitations of scientific endeavors. Scientists should prioritize respecting humanity and maintaining an attitude of reverence for other living beings (Compendium of the Social Doctrine of the Church, 2006). Scientists should employ empirical science for the common good, as stated in the Catechism of the Catholic Church, which firmly asserts that human power has boundaries and causing needless suffering or death to animals is contrary to human dignity (Gregg, 2006).

In addition, in 2023, it has been published Pope Francis' new Apostolic Exhortation Laudate Deum on the climate crisis. The Holy Father addresses the situation of climate change in Laudate Deum ("Praise God") because "a human being who pretends to take the place of God becomes the worst danger to himself" (LD 73) after the Encyclical Letter Laudato si' promulgated on the Solemnity of Pentecost 2015. The Apostolic Exhortation Laudate Deum, directed at individuals of goodwill, consists of six chapters. In the final chapter titled "Spiritual Motivations," the Holy Father urges individuals from various religious backgrounds to act. Additionally, he emphasizes to Catholics that their faith obliges them to protect God's creation by respecting the laws of nature and appreciating the beauty and abundance of God's handiwork.

Furthermore, health professions, such as nursing, consider global care as part of their daily work in their professional code of ethics: Nurses value health care as a human right, affirming the right, nurses lead or contribute to sound health policy development, nurses contribute to population health and work toward the achievement of the United Nations Sustainable Development Goals and nurses collaborate and practice to preserve, sustain, and protect the natural environment, and are aware of the health consequences of environmental degradation, e.g., climate change. They advocate for initiatives that reduce environmentally harmful practices to promote health and well-being (International Council of Nurses, 2021). Therefore, considering ecospirituality as part of health care will contribute to providing more global, fair, and humanized care.

### Limitations

This study has important strengths. This review has allowed the authors to update knowledge about ecospirituality and identify gaps in the evidence base and the possibility of raising new research questions. However, this study has several limitations that should also be taken into account. The first is that the search was carried out in only three databases (PubMed, Scopus, and WOS), which has prevented access to all the articles that may have been carried out in relation to the research question posed. Only studies on ecospirituality were included in this review. Besides, there is also a language restriction, and articles published in other laguanges were not included. Finally, authors set our date limit to the past 5 years aiming to present an update panorama of the field. Therefore, studies published before 2018 were not included.

## Conclusions

A new perspective in our vision of the world such as ecospirituality will provide humanity with the necessary keys to improve environmental health and thereby achieve global health. To understand ecospirituality, humanity must keep in mind the indigenous way of life, which is the clear example to follow to achieve environmental health and global health. Ecospirituality evokes pro-environmental behaviors and increases mental health. Ecospirituality leads toward a healthier environment, and since the environment is directly related to health, there is also an improvement in overall health. It is necessary for the health sector to use a biopsychosocial model for its care, where work is done in the spiritual sphere and through it strategies based on ecospirituality are applied, such as sociocultural integration and ecocounseling.

## Appendix 1

### Quality Assessment of Included Studies

**Table:**

*Mixed Method Studies*						
Study	Total score	Is there an adequate rationale for using a mixed methods design to address the research question?	Are the different components of the study effectively integrated to answer the research question?	Are the outcomes of the integration of qualitative and quantitative components adequately interpreted?	Are the divergences and inconsistencies between quantitative and qualitative results adequately addressed?	Do the different components of the study adhere to the quality criteria of each tradition of the methods involved
Nawrath et al. (2022)	5/5	Yes	Yes	Yes	Yes	Yes
*Qualitative study*						
Study	Total score	Is the qualitative approach appropriate to answer the research question?	Are the qualitative data collection methods adequate to address the research question?	Are the findings adequately derived from the data?	Is the interpretation of results sufficiently substantiated by data?	Do the different components of the study adhere to the quality criteria of each tradition of the methods involved
Bellehumeuret al. (2022)	3/5	Yes	Yes	Yes	No	Cannot tell
Cloud and Redvers (2023)	3/5	Yes	Yes	Yes	No	Cannot tell
Denis et al. (2022)	4/5	Yes	No	Yes	No	Yes
Hategan (2021)	5/5	Yes	Yes	Yes	Yes	Yes
Keaulana et al. (2021)	5/5	Yes	Yes	Yes	Yes	Yes
Lestar and Böhm (2020)	3/5	Yes	Yes	Yes	No	Cannot tell
Nalau et al. (2018)	3/5	Yes	Yes	Yes	No	Cannot tell
Oberholzer-Dent et al. (2023)	3/5	Yes	Yes	Yes	No	Cannot tell
Skoko et al. (2021)	3/5	Yes	No	Yes	Yes	Cannot tell
*Quantitative Studies*						
Study	Total score	Is the sampling strategy relevant to address the research question?	Is the sample representative of the target population?	Are the measurements appropriate?	Is the risk of nonresponse bias low?	Is the statistical analysis appropriate to answer the research question?
Kras and Keenan (2020)	5/5	Yes	Yes	Yes	Yes	Yes
Oliveira et al. (2022)	5/5	Yes	Yes	Yes	Yes	Yes
Vitorino et al. (2018)	5/5	Yes	Yes	Yes	Yes	Yes
Walls et al. (2022)	5/5	Yes	Yes	Yes	Yes	Yes

Yes—1; no—0; and cannot tell—0
