# Correction: Ecospirituality and Health: A Systematic Review

**DOI:** 10.1007/s10943-025-02277-8

**Published:** 2025-02-24

**Authors:** Rocío de Diego-Cordero, Alicia Martínez-Herrera, Manuel Coheña-Jiménez, Giancarlo Lucchetti, José Miguel Pérez-Jiménez

**Affiliations:** 1 Research Group PAIDI‑CTS 969 Innovation in HealthCare and Social Determinants of Health, Department of Nursing, Faculty of Nursing, Physiotherapy and Podiatry, University of Seville, Seville, Spain; 2 Department of Nursing, Faculty of Nursing, Physiotherapy and Podiatry, University of Seville, Seville, Spain; 3 Research Group PAIDI-CTS589: Advances in Podiatric Surgery, Department of Podiatry, Faculty of Nursing, Physiotherapy and Podiatry, University of Seville, C/Avenzoar, 6, 41009 Seville, Spain; 4 School of Medicine, Federal University of Juiz de Fora, Juiz de Fora, Brazil; 5 Institute of Biomedicine of Seville (IBiS), Department of Nursing, Faculty of Nursing, Physiotherapy and Podiatry, University of Seville, Seville, Spain; 6 Institute of Biomedicine of Seville (IBiS), Universitary Hospital Virgen Macarena, University of Seville, Seville, Spain

**Correction to: Journal of Religion and Health (2024) 63:1285–1306 ** 10.1007/s10943-023-01994-2

In this article, the author name Giancarlo Lucchetti was incorrectly written as Giancarlo Luchetti. The original article has been corrected.

Incorrect name: **Giancarlo Luchetti**

Correct name: **Giancarlo Lucchetti**

